# Characterization of the mechanical properties of qPlus sensors

**DOI:** 10.3762/bjnano.4.1

**Published:** 2013-01-02

**Authors:** Jan Berger, Martin Švec, Martin Müller, Martin Ledinský, Antonín Fejfar, Pavel Jelínek, Zsolt Majzik

**Affiliations:** 1 Institute of Physics, Academy of Sciences of the Czech Republic, Cukrovarnicka 10, 162 53, Prague, Czech Republic

**Keywords:** AFM, Cleveland’s method, cross talk, force, qPlus, stiffness, STM, thermal noise, tuning fork

## Abstract

In this paper we present a comparison of three different methods that can be used for estimating the stiffness of qPlus sensors. The first method is based on continuum theory of elasticity. The second (Cleveland’s method) uses the change in the eigenfrequency that is induced by the loading of small masses. Finally, the stiffness is obtained by analysis of the thermal noise spectrum. We show that all three methods give very similar results. Surprisingly, neither the gold wire nor the gluing give rise to significant changes of the stiffness in the case of our home-built sensors. Furthermore we describe a fast and cost-effective way to perform Cleveland’s method. This method is based on gluing small pieces of a tungsten wire; the mass is obtained from the volume of the wire, which is measured by optical microscopy. To facilitate detection of oscillation eigenfrequencies under ambient conditions, we designed and built a device for testing qPlus sensors.

## Introduction

The invention of scanning tunneling microscopy [1] and atomic force microscopy (AFM) [2] opened new horizons in characterization and modification of surfaces and nanostructures. STM is routinely used nowadays as a standard technique to characterize and modify objects at the atomic scale. However, its application is limited only to conductive samples as the tunneling current flowing between a probe and a sample is employed as the feedback signal.

This limitation was surpassed by AFM techniques, which are essentially based on force detection acting between the tip and the sample. In principle, this approach can be adapted to any arbitrary sample, independently of its conductivity properties. Consequently AFM techniques have found many applications across different scientific fields, including biology, chemistry and physics. In particular, noncontact atomic force microscopy [3] (nc-AFM) has developed into a powerful technique for imaging with true atomic resolution [4–5], chemical sensitivity [6–8] or for performing single atom manipulation [9–11] on all types of surfaces, including insulators.

Shortly after the invention of AFM and STM, the first attempt to combine static AFM and STM measurements was made by Dürig et al. in 1986 [12]. A few years later, combined nc-AFM/STM using a Si cantilever was reported for the first time (see [13]), showing the capability to record simultaneous STM and AFM signals with atomic resolution on a metal surface [14]. At the same time F. J. Giessibl introduced so-called qPlus sensors [15], which allow simultaneous acquisition of the tunneling current and the forces with a small oscillation amplitude. This method increases substantially the sensitivity to the tunneling current signal comparing to traditional Si cantilevers. This new approach opens new possibilities in the characterization of surfaces and nanostructures on the atomic scale (see, e.g., [16]). Not surprisingly, qPlus sensors have become frequently used for noncontact measurements nowadays (see, e.g., [7,9,17–21]).

The heart of the qPlus-based AFM/STM microscope is a tuning fork with one prong fixed and the other, with a metallic tip positioned at the very end, freely oscillating. The variation of the resonant frequency of the prong reflects directly the interaction of the sensor with the sample. At the same time, the presence of a metallic tip allows simultaneous acquisition of the average tunneling current flowing between tip and sample when the bias voltage is applied. In principle, the force acting between the tip and sample might be accessible from the detected frequency modulation. However, a reliable estimation of the measured force depends on several factors, among them the proper calibration of the mechanical properties (stiffness) of the sensor.

Originally all mass-produced tuning forks are tuned to the same frequency by laser trimming. However, it has been shown that the sensor fabrication process may strongly alter the stiffness [22–23]. The value of the stiffness can also be influenced by fixing the tip on the prong and by the gluing process. Furthermore, it has been shown that a slight shortening of the oscillating prong may be beneficial for improving electrical sensitivity of the sensor [20]. What is more, when the tunneling current is collected on the tip, an additional wire, usually gold, needs to be attached for STM measurements in order to avoid any interference between the tunneling current and deflection channels [20]. This wiring may have a certain impact on the mechanical properties of the sensor. Therefore to ensure proper estimation of detected forces the mechanical properties of each sensor should be calibrated carefully.

In the past, several methods have been developed to estimate the stiffness of sensors [24]. The first class of methods uses the variation of the resonant frequency before and after the addition of some small mass to the end of the prong. One example of this method is the added-mass (Cleveland’s) method [25]. The second method estimates stiffness from the bending of the sensor as a function of the applied force [26]. The third method uses the thermal motion of the sensor to estimate the stiffness (for details see [27]). Additionally, the stiffness can be calculated directly from the elastic properties of the sensor [28]. All methods have some advantages and limitations, as we will discuss later.

The aim of this paper is to compare and critically discuss the following methods for estimating the stiffness of qPlus sensors: (i) the added-mass method; (ii) thermal excitation; and (iii) a method based on the continuum theory of elasticity. In particular we will estimate and compare the stiffness of several home-built and commercial qPlus sensors using different methods. We will also briefly describe a simple testing device for reliable detection of the mechanical properties of sensors under ambient conditions.

## Experimental

### Sensors

In this work, we used two types of sensors: commercial (Omicron–Oxford instruments) (com) and home-built (hb) sensors. The commercial sensors have a length of ~2.4 mm and the tip is placed on the side of the prong usually around 0.05 mm from the end. No additional wire is used in this design for the tip connection.

**Figure 1:**
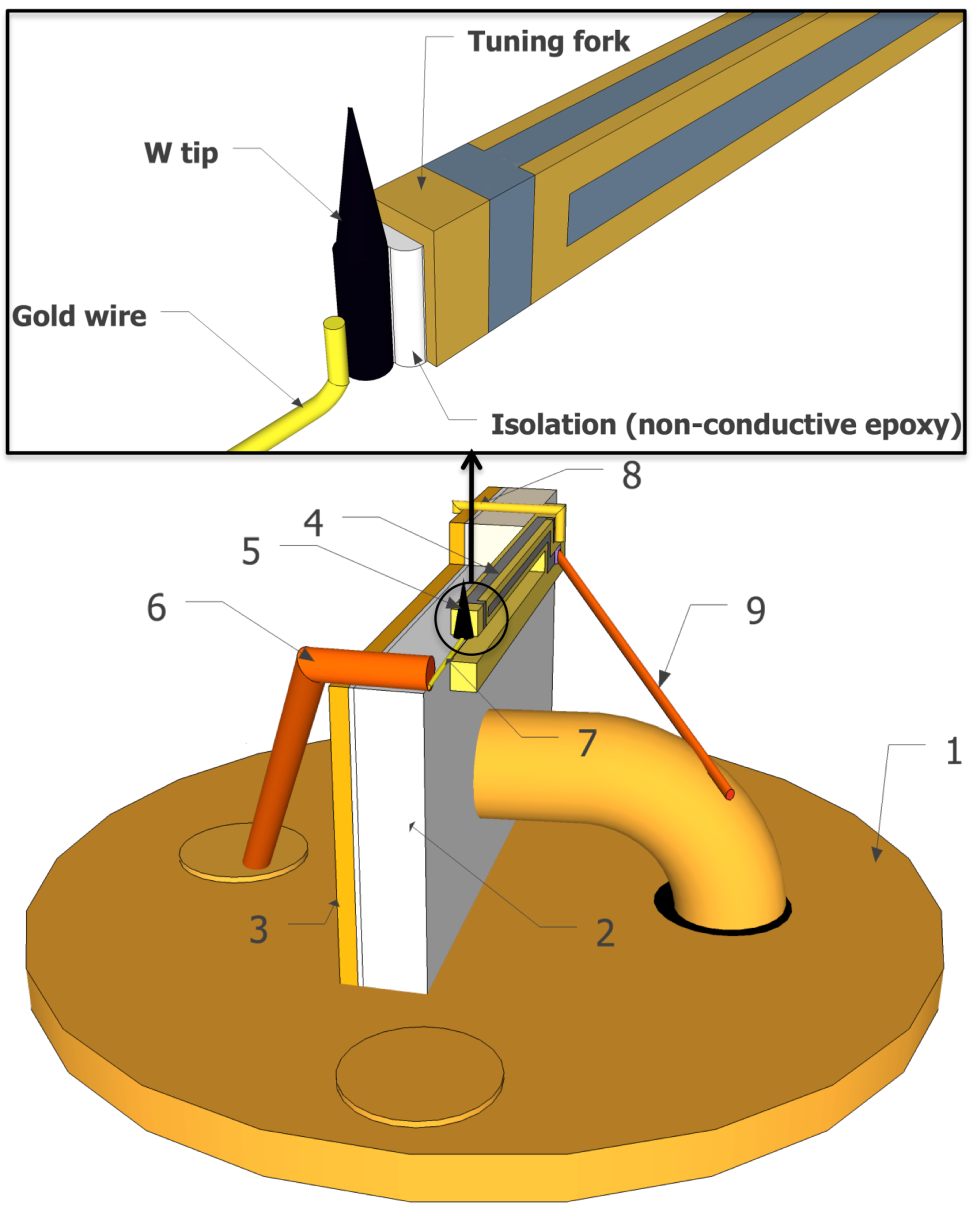
Home-made qPlus sensor consists of the following parts: (1) base stage (from Omicron–Oxford instruments), (2) ceramic plate, (3) copper shield that is glued to the ceramic plate (4) (shortened) tuning fork, (5) tip, (6) copper wire for supporting the gold wire, (7) gold wire used for making connection to the tip, (8) gold wire providing connection between copper shield and the shielding electrode, (9) gold (or copper) wire for collecting the deflection signal [20]. Detail shows the fixing of the tip to the prong and gold wire for collecting the tunneling current.

The home-built qPlus sensors were built from commercially available tuning forks from Micro Crystal, in the SMD package MS1V-T1K. The original tuning forks were shortened in order to reach higher sensitivity (charge produced by deflection), which allows us to operate with lower amplitudes [20,29]. The schematic figure of the sensor and a detail of a tip mounting is shown in Figure 1. Ceramic plates were used as a support for tuning forks. One side of these plates was covered by a very thin copper layer for shielding [20]. The tip itself is connected to one of the pins of the sensor by a combination of gold (ø = 25 μm) and copper (ø = 500 μm) wire. In order to minimize unnecessary vibrations the copper wire is fixed to the copper shield by nonconductive epoxy (Torr seal). As a result, only the thin gold wire is allowed to vibrate with the motion of the prong. The gold wire is attached to the end of the prong by the same nonconductive epoxy as used before and, as the last step during the sensor construction, the tip is carefully mounted directly to the gold wire by a conductive epoxy (EPO-TEK H21D) in such a way that there is no additional electrical connection to the rest of the prong. Tips are etched from 0.125 μm tungsten wire in 2 M solution of NaOH. We use the drop-off method for etching our tips, thus the final tips have the shape of a droplet and they are between 200 and 300 μm long.

#### Testing device for measurements in ambient conditions

**Figure 2:**
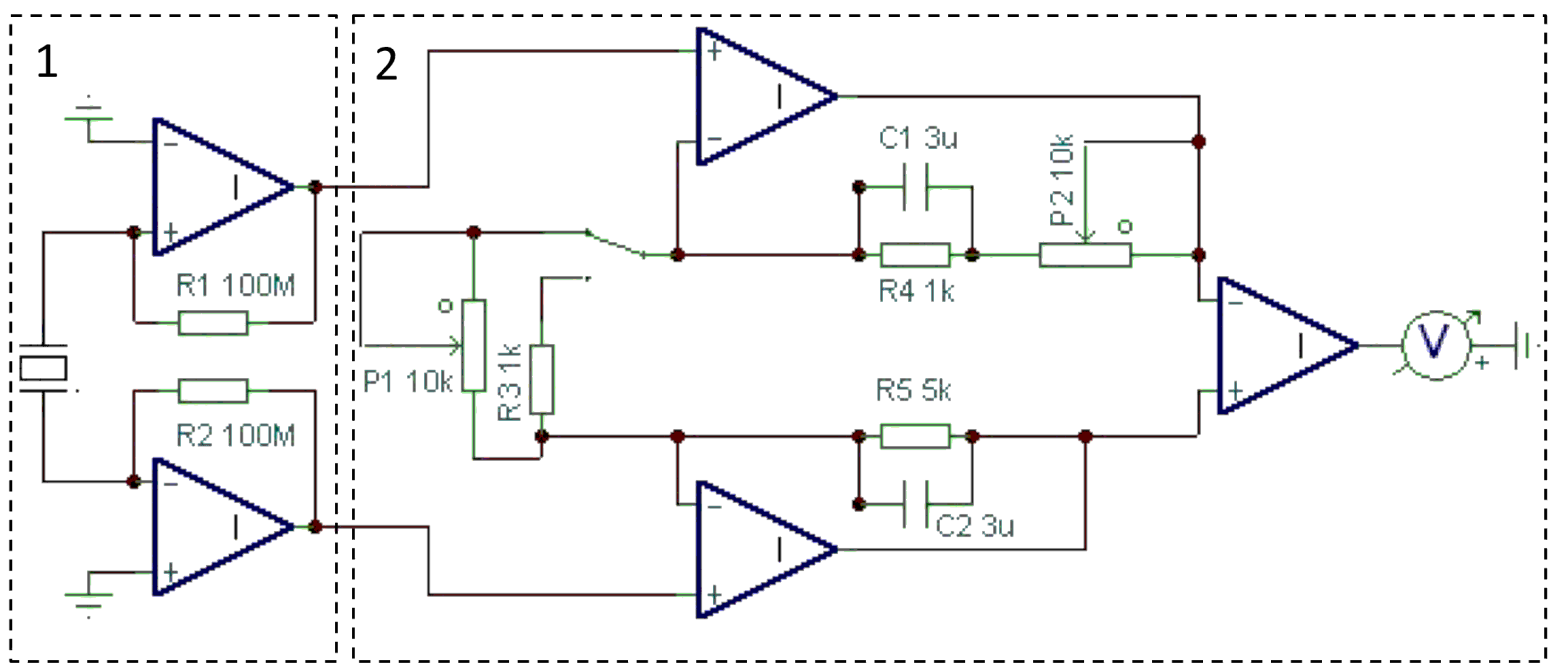
Two-stage amplifier used for testing qPlus sensors. The first stage works as a charge amplifier in the frequency range of our tuning forks. The second stage is a differential amplifier, which allows us to improve the signal-to-noise ratio [30] and can eliminate the driving signal, which is coupled by stray capacitances, from the deflection channel.

Cleveland’s method requires detection of an eigenfrequency. To facilitate these measurements, we constructed a simple device for testing the mechanical properties of qPlus sensors under ambient conditions. The tester has two amplification stages as shown in Figure 2. The first stage contains two current-to-voltage converters (IVC) that are used to generate voltage signals from the small currents produced by two electrodes of the fork during its sinusoidal motion. OP 111 operational amplifiers (OPA) in a TO 99 package were used as IVCs with 100 MΩ feedback resistors. In order to gain the maximum performance of the device, the inputs of amplifiers were brought as close as possible to the outputs of the sensor: the lengths of the connecting wires were about 1 cm only. Furthermore, the SMD-packed feedback resistor was mounted directly between the input and the output legs minimizing the length of wiring to a few millimetres. By this construction the input and the parasitic capacitance of the feedback resistor could be minimized. Note that nowadays the old OP 111 can be replaced by a faster, less noisy OPA (e.g., OP 637, etc.). However in the frequency range of our interest, OP 111 performed satisfactorily.

The second stage is a differential stage, in which the output voltage of the first stage can be optionally enhanced up to 103 times. Due to the electrode configuration of the prong, the signals from the first stage are in inverse phase with respect to each other. Therefore the application of a differential amplifier leads to a better signal-to-noise ratio [30]. Furthermore, the two OPAs in the first stage were mounted in a way that the stray capacitances are tuned to be nearly the same. By this design, the driving signal is coupled by stray capacitances almost identically (phase and amplitude) to each input of the OPAs. Consequently this unwanted component becomes almost completely nulled out by the differential stage. This procedure can be applied to minimize coupling from other sources, e.g., bias modulation used for Kelvin probe measurements.

A piezo tube is used for mechanical excitation. Apart from the rubber legs of this small instrument there is no additional vibration isolation. Finally, SPECS Nanonis OC-4 PLL was used for data acquisition.

#### UHV measurements

The measurements requiring precise vibration and sound isolation were carried out in UHV on an Omicron VT XA qPlus AFM/STM at room temperature (RT) at a base pressure below 1 × 10^−10^ mbar. The resonant frequency *ν* was determined by using the same PLL as for the ambient measurement. A high-quality PC sound card ASUS Xonar Essence ST in combination with a free FFT software Spectrum Lab (Audio Signal Analyser) was used to record thermal-noise density spectra.

## Results and Discussion

We carried out a series of measurements of sensors with prong lengths varying from 1.8 to 2.4 mm. Due to the variation of the length and of the mass of the attached tips, the resonant frequency altered between 25 and 60 kHz. For commercial sensors, the tip was attached on the side of the prong, effectively shortening the tuning fork. Consequently two values of stiffness can be considered; one taking into account the whole length of the prong, and another that considers the length defined by the tip mounting position (effective stiffness). Compared to the case of real physical shortening (cutting), the remaining part of the prong adds extra mass, and therefore it has certainly a negative effect on the resonant frequency. The interesting question is whether this kind of tip attachment has a significant effect on the stiffness measurement. In the following part of the text, the effective stiffness will be shown in brackets right after the value corresponding to the total length.

### Stiffness calculation from continuum theory of elasticity

The tuning fork can be considered as a pure prism with a rectangular cross section. Therefore the stiffness of the tuning fork can be expressed by using the continuum theory of elasticity [31] as follows:

[1]
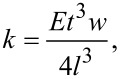


where *E* is Young’s elastic modulus (for quartz *E* = 78.7 GPa), *t* is the thickness (0.214 mm), *w* is the width (0.126 mm), *l* is the length of the prong, and *k* is the stiffness of the tuning fork. In order to determine the stiffness in this work, the length, which is the only variable, needs to be known. All remaining parts can be considered as constants because (i) we assume the Young’s modulus to be constant for quartz tuning forks; and (ii) we found from repeated measurements that variations in *t* and *w* are negligible (less than ±2 μm) for our purposes.

The dimensions of the prong were determined by an optical microscope Leica DM LM prior to insertion into UHV. The horizontal resolution of this microscope with objective Leica N PLAN L 50× (NA 0.5) is at least 2 μm. The measured lengths and calculated stiffness values are listed in Table 1. By using the minimal resolution for the microscope, the error of measuring the length of the prong is below 0.1% and errors of the thickness and the width are below 1.6%. The final precision of this method is in the range of 5%.

**Table 1:** Measured lengths of tested sensors and calculated stiffnesses. In the case of the commercial sensor, the length defined by the tip mounting position and the resulting effective stiffness is shown in brackets. Estimated error is in the range of 5%.

Sensor	hb_1_	hb_2_	hb_3_	hb_4_	com

*l* [mm]	1.853	1.842	1.816	2.314	2.4 (2.35)
*k* [N/m]	3818 ± 83	3887 ± 78	4056 ± 101	1943 ± 56	1757 ± 43 (1872 ± 43)

### Added-mass method

Cleveland et al. presented a very intuitive way for precise determination of the stiffness, which is based on the measurement of the change in the resonant frequency. This change is induced by loading an extra mass to the lever, *M*, which is relatively small compared to the total mass of the oscillator, m*. The effect of *M* on the resonant frequency *ν* can be estimated as follows:

[2]
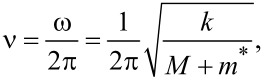


where ω is the angular velocity. The Equation 2 can be rewritten in a more usable form:

[3]
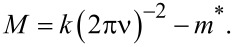


From Equation 3 we directly see that the loaded mass *M* is linearly proportional to (2*πν*)^−2^. Therefore the dependence of *M* versus (2*πν*)^−2^ should be linear, with a slope corresponding to the stiffness *k*.

Consequently the stiffness *k* can be directly calculated from the values of resonant frequencies before (*ν*_0_) and after (*ν*_1_) loading the lever with the mass *M*:

[4]
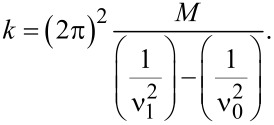


Because the frequency can be measured with very high precision, the precision of the method relies mainly on the mass measurement. For silicon cantilevers, several ideas have been proposed for how to load them with known masses (see [25,32–33]). In our case, the large tuning forks make the task somewhat easier because they can support heavier objects. For this reason, we introduce a method that does not require any expensive microbalance scale or other types of high precision instrument. In this method, we fix a small piece of tungsten wire as close as possible to the tip. The mass of the load was estimated from its dimensions measured with the optical microscope and afterwards calculated using the bulk density of tungsten ρ = 19.3 g/cm^3^.

**Figure 3:**
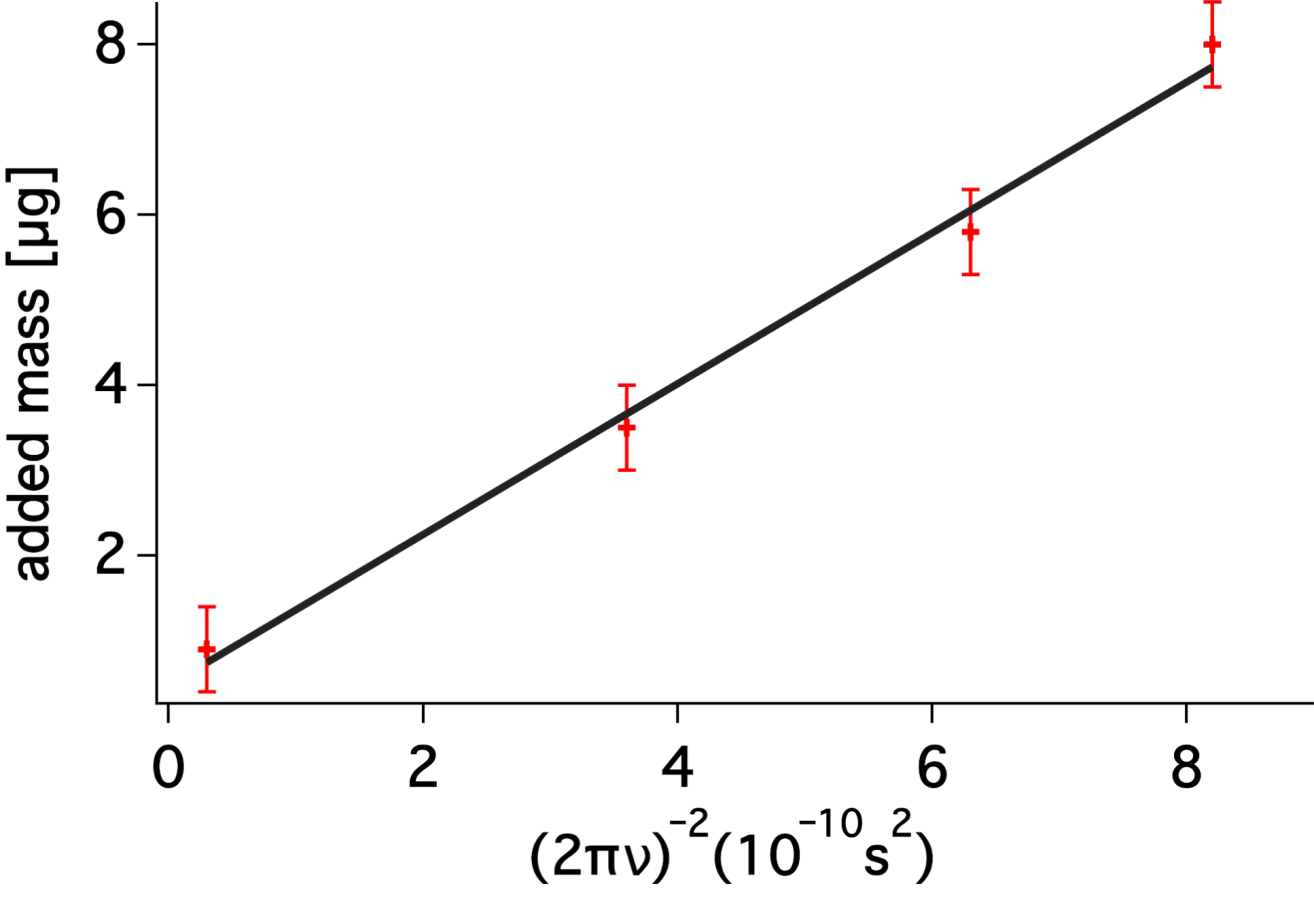
A plot of added mass versus (2*πν*)^−2^(10^−10^s^2^) for a single commercial sensor. A simple linear regression of the measured data gives a stiffness, in this case the slope of linear fit, of 1753 ± 135 N/m.

The amount of added mass must be chosen carefully as overloading the tuning fork may lead to anharmonicity, and consequently Equation 3 will be not valid anymore. In order to be sure that overloading does not occur, we added several loads to a single sensor and the resonant frequency was measured for each added mass. As it is clearly visible in Figure 3 the linearity is still kept and thus Equation 3 and Equation 4 remain valid in the range of the given masses.

**Figure 4:**
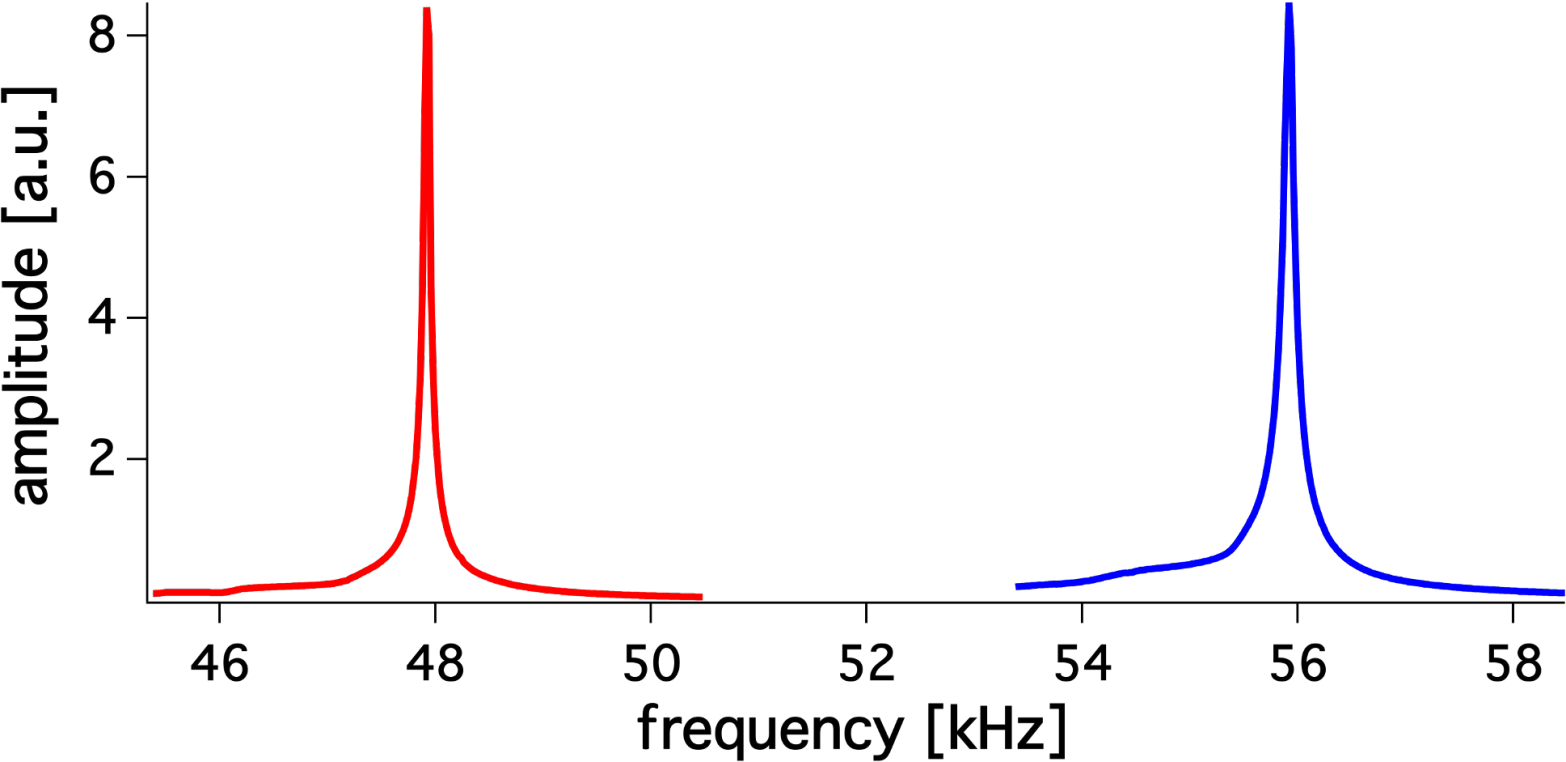
Graph shows two resonant curves. The blue curve corresponds to an unloaded tuning fork with a resonance frequency of 55872 Hz. The red one refers to the same fork end-loaded with 11.43 μg. The resonance frequency shifts to 47787 Hz.

Naturally, the tungsten wire needs to be fixed in a rigid way. For this purpose, we used the Torr seal^®^ epoxy, which was used for sensor construction as well. On the one hand, experiments showed that the adhesion force between tuning fork and glue was strong enough to hold the tungsten wire during the measurement. On the other hand, the surface of the tuning fork is smooth. Therefore the glue can be easily removed after measuring, without damaging the sensor and voiding its subsequent application. Unquestionably, the glue adds some extra mass that we do not take into account in our method. However, the amount of the glue used to fix the extra wire causes a change in frequency that is negligible compared to the large frequency shift caused by the tungsten wire loading. Figure 4 gives a typical example of the change in the resonant frequencies after adding an extra load.

Results obtained by the added mass method for different sensors are summarized in the Table 2. Lengths and diameter of wires used as added masses were about 300 μm and 50 μm, respectively. The optical microscope utilized in this work has a resolution of about 2 μm, from this we estimate an error of the length measurement less than 1% and for the diameter of ≈8%. Consequently, the estimation of the added mass has a maximal error of about 11%. Because the spring constant depends on the added mass linearly and the error arising from the determination of frequencies is negligible, the estimated maximal error of the stiffness from Cleveland’s method is also around 11%.

**Table 2:** Eigenfrequencies of tuning forks before (*ν*_0_) and after (*ν*_1_) loading of an extra mass *M* to the prong. In the case of the commercial sensor, the length defined by the tip mounting position and the resulting effective stiffness is shown in brackets. Sensor hb_3_ was measured with and without gold wire (with the same added mass). These values show that the influence of the gold wire is minor for shortened tuning forks.

Sensor	hb_1_	hb_2_	hb_3_	hb_3_–w/o wire	hb_4_	com

*ν*_0_ [Hz]	55872	48596	57030	58200	25929	25573
*ν*_1_ [Hz]	47787	43203	46283	46997	23728	23581 (23286)
*k* [N/m]	3835 ± 211	3846 ± 218	3945 ± 217	3991 ± 220	1857 ± 105	1782 ± 101 (1829 ±103)
*M* [μg]	11.43	10.96	15.94	15.94	13.60	12.17 (14.62)

### Thermal excitation

The harmonic oscillator in equilibrium with its environment fluctuates in response to the thermal noise. The prong of the tuning fork is assumed to behave like a simple harmonic oscillator. The normal stiffness of the qPlus sensor can be related by the equipartition theorem to its thermal energy during vibration, leading to the relationship

[5]
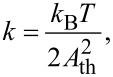


where *A*_th_ is the amplitude of the thermal motion of the free prong, *k*_B_ is the Boltzman constant (*k*_B_ = 1.38 × 10^−23^ J·K^−1^), *T* is the temperature of the system (in our case it is RT) and *k* is the stiffness of the tuning fork. This method can be used for stiffness measurement if the oscillation is only thermally excited without any additional excitation (for instance mechanical vibration). This method is the only one mentioned in this paper that is able to estimate the spring constant during the course of an UHV AFM experiment. Generally, UHV AFM/STM instruments are able to reach a high level of vibrational isolation, which is needed to minimize extra mechanical excitation from outside of the system.

The square of the output voltage of the thermally exited tuning fork can be expressed as

[6]
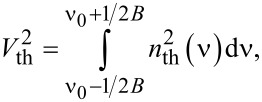


where *n*_th_ is the thermal noise density and *B* is the bandwidth [34]. In Equation 6 the range of the measurement determined by *B* has to contain all the resonant features. In principle, as long as this rule holds, the bandwidth should not play any role in the measurement. If the peak width is much larger than the bandwidth, inaccurate results are expected.

There is another factor that has to be considered: In Equation 6 the thermal noise density can be obtained from the power noise density, 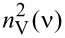, by subtracting the electrical noise density 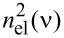 from it, thus 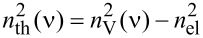. The electrical noise density, 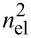, should have white-noise character around the resonance, as a consequence, 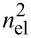 does not depend on the frequency. This requirement is usually well fulfilled around the resonant frequency of our homemade sensors.

In order to be able to evaluate the physical magnitude of the oscillation amplitude, the conversion factor (*C* = *V*_th_/*A*_th_) needs to be determined. *C* was estimated during dynamic STM measurement on a clean Si(111)-(7 × 7) surface with a precision of ≈10%. It is worth noting that, as the amplitude *A*_th_ is represented in Equation 5 as a square value, the error in the stiffness measurement will be two times higher than the error in the amplitude.

Stiffness values that were calculated from the thermal noise of the sensor are summarized in Table 3.

**Table 3:** Stiffness values for home-built sensors measured by thermal-excitation method. Commercial sensors were not tested, because our qPlus system is modified for our home-made sensors only.

Sensor	hb_1_	hb_2_	hb_3_	hb_4_

*k* [N/m]	3650 ± 369	3702 ± 367	3872 ± 382	1779 ± 179

### Comparison of the methods

We found good agreement between calculated stiffness values using the three methods, as can be seen from a direct comparison in Table 4. The variation in the calculated stiffness values stays within the 10% range for both the continuum theory of elasticity and Cleveland’s method; and within 20% range for the thermal-excitation method (see Table 4). Surprisingly, we did not observe a systematic error between results provided by the added-mass method and calculated stiffness values from the continuum theory of elasticity. This finding suggests that the thin gold wire attached to the end of the prong has only a minor effect on the overall stiffness. In the case of a straight Au wire, the stiffness can be obtained from

[7]
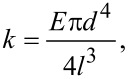


**Table 4:** Summary of stiffness values obtained by the introduced methods.

Sensor	*l* [mm]	*k* [N/m]		

		Continuum theory of elasticity	Cleveland’s method	Thermal excitation

hb_1_	1.802	3818 ± 83	3835 ± 211	3650 ± 369
hb_2_	1.769	3887 ± 78	3846 ± 218	3702 ± 367
hb_3_	1.816	4056 ± 101	3945 ± 217	3872 ± 382
hb_3_–w/o wire	–	–	3991 ± 220	–
hb_4_	2.314	1943 ± 56	1857 ± 105	1779 ± 179
com	2.400 (2.385)	1757 ± 43 (1872 ± 43)	1782 ± 101 (1829 ± 104)	–

where *d* is the diameter, *l* is the length of the wire and *E* is the Young’s modulus (79 GPa for Au). The negligible role of the wire is mainly due to the relatively small diameter (25 μm) of the Au wire. In our case, the typical length of the Au wire is ≈500–600 μm. Using Equation 7, we obtain the stiffness of the gold wire ≈200–160 N/m. This value is much lower than the stiffness of the tuning fork itself (especially of shortened tuning forks). To test the negligible effect of the gold wire on the sensor stiffness in more detail, we repeated the added-mass measurement after the wire had been removed (see Table 4, sensor hb_3_ and hb_3_–w/o wire). From the data we obtained it is evident that the calculated stiffness does not show any significant changes.

From Table 4 we see that the thermal-peak method systematically underestimates the stiffness compared to two other methods. We attribute this error to additional (e.g., mechanical) excitation presented during the measurements. It was shown by Welker et al. [34] that the mechanical noise becomes dominant at low temperature, which would imply a very large error in stiffness calculations based on thermal-noise analysis. However, we performed our calibration procedure at RT, where the thermal excitation is much larger and the quality factor of the tuning fork is also significantly lower (in our case *Q* ≈ 1500–4500). Thus the tuning fork is less responsive to mechanical vibration, although it still cannot be omitted completely. The piezo scanner was connected to the ground during the thermal-noise measurement, therefore we can rule out the possibility of additional excitation by electrical noise.

Finally, we would like to address the question of how the precision of the stiffness would affect experimental force measurements. The force can be expressed by using the Sader formula [35] as follows

[8]
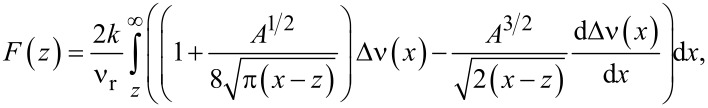


where Δ*ν* is the frequency shift, *ν*_r_ is the resonant frequency, *k* is the stiffness of the sensor, *A* is the amplitude of oscillation, *F* is the tip–surface interaction force, *x* is the tip–surface distance, and *z* being the closest tip approach towards the sample.

From Equation 8 it is clear, that an error in the stiffness leads to a proportional but systematic error in the force measurement. In the large amplitude limit, there is a second dominant source of errors in quantitative force analyses, namely the amplitude calibration (see Equation 8).

The other two methods do not depend on the amplitude calibration. They depend only on size measurements, which can be calibrated with very high accuracy by using optical or electron microscopes. Therefore, we suppose that the overall error in the force measurement is smaller with these methods. Especially with the added-mass method because this can take into account other influences on the stiffness such as improper gluing during sensor construction [22]. We have to note here that in the case of our home-built sensors neither the gluing nor the length of the tip seem to have a strong effect on the stiffness. However, due to the lack of usability of the original, commercial sensors, we do not have enough samples to be able to contribute to the previously reported statistics.

Finally the main benefit of the thermal-noise measurement is that it can be performed any time during the actual AFM measurement, and at least at RT we have seen quite a good agreement with the other two methods.

## Conclusion

In this paper we applied and compared three different methods for the estimation of the stiffness of qPlus sensors. Our analysis showed that all three methods give very similar results varying within a range of 10% or 14% for the thermal-excitation method. Surprisingly, the added-mass (Cleveland’s) method gave very similar results to calculated values from the continuum theory of elasticity. This finding suggests that a gold wire does not strongly alter the stiffness. Furthermore, we have proved that the stiffness can be obtained with reasonably small error from the thermal noise measurement even at RT.

In addition we designed and built a device for testing qPlus sensors. It can be used for testing sensors before inserting into the chamber and to obtain information about mechanical properties of sensors such as the resonant frequency, quality factor and stiffness. We also discussed a fast and cost-effective way to perform the added-mass method under ambient conditions. This method is based on adding small pieces of tungsten wire whose mass was determined from the volume of the wire. The wire can be easily removed after the measurement without destroying the sensor.
